# Low-entry-barrier point-of-care testing of anti-SARS-CoV-2 IgG in the population of Upper Austria from December 2020 until April 2021—a feasible surveillance strategy for post-pandemic monitoring?

**DOI:** 10.1007/s00216-022-03966-z

**Published:** 2022-02-28

**Authors:** Christian Doppler, Michael Feischl, Clara Ganhör, Spela Puh, Marina Müller, Michaela Kotnik, Teresa Mimler, Max Sonnleitner, David Bernhard, Christian Wechselberger

**Affiliations:** 1 Division of Pathophysiology, Institute of Physiology and Pathophysiology, Medical Faculty, Johannes Kepler University Linz, Krankenhausstrasse 5, 4020 Linz, Austria; 2 Institute for Analysis and Scientific Computing, TU Vienna, Vienna, Austria; 3 Cardiac Surgery Research Laboratory, Department of Cardiac Surgery, Medical University of Vienna, Vienna, Austria; 4 Genspeed Biotech GmbH, Rainbach, Austria

**Keywords:** SARS-CoV-2, COVID-19, Rapid test, Point-of-care, Immunoglobulin, Surveillance

## Abstract

Already at the very beginning of the COVID-19 pandemic, an extensive PCR and antigen testing strategy was considered necessary and subsequently also proved successful in order to limit the spread of severe acute respiratory syndrome coronavirus 2 (SARS-CoV-2) infections on international and national levels. However, equally important will be the continuous monitoring of the seroprevalence status of populations from defined regions to detect—in a timely manner—any recurrence of infections or an eventual decline in antibody levels of vaccinated individuals, especially in the emerging post-pandemic situation. The aim of this study was to estimate the prevalence of SARS-CoV-2-specific immunoglobulin G antibodies in the federal state of Upper Austria (Austria) during the period of December 2020 until April 2021. To achieve this goal, we have analyzed anonymized data on the immune status of self-referral volunteers that have been determined at local pharmacies through a low-entry-barrier point-of-care analysis approach. The seroprevalence values for immunoglobulin type G antibodies against SARS-CoV-2 antigens obtained by rapid diagnostic testing on peripheral blood from volunteers reflect the current population-based estimates reported in the literature as well as the positivity rates detected by PCR-screening analyses. In conclusion, broad-based monitoring of IgG antibodies by means of a point-of-care testing network represents a valuable tool to assess the current immune situation within regionally defined populations.

**Graphical abstract:**

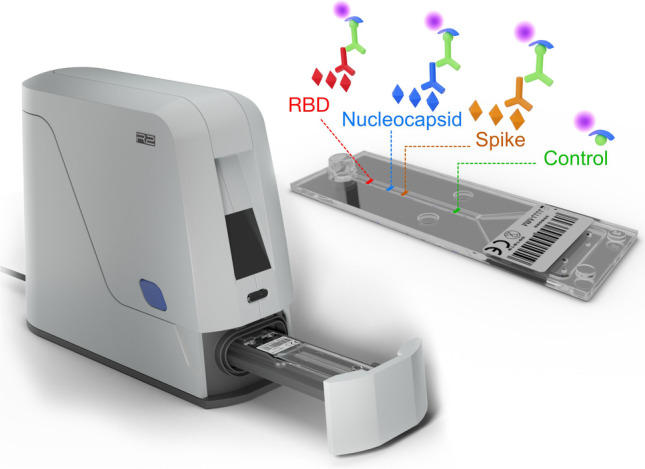

## Introduction

Since December 31, 2019, and as of week 2021–37, 229,415,774 cases of coronavirus disease 2019 (COVID-19; in accordance with the applied case definitions and testing strategies in the affected countries) have been reported, including 4,699,359 deaths (https://www.ecdc.europa.eu/en/geographical-distribution-2019-ncov-cases). In Austria, the first case of COVID-19 was reported on February 26, 2020. On March 16, 2020, a general lockdown was implemented across the country, along with specific public health measures aimed at reducing the spread of infections. Nonetheless, by September 27, 2021, 732,259 cases of COVID-19 and 10,736 associated deaths have been reported (https://covid19-dashboard.ages.at/). Starting with December 27, 2020, highly effective vaccination regimes have been deployed in Austria, and as of September 27, 2021, already 63.88% of the population have received at least one dose of vaccine (https://info.gesundheitsministerium.at/). However, numbers of fatalities will continue to rise in the near future, and keeping an eye on the rate of new infections still represents a major task. Due to the high transmissibility of SARS-CoV-2 also in asymptomatic patients, diagnosis relying solely on symptoms and contact tracing alone has proven difficult already during the early days of the recent COVID-19 pandemic. This will also be a serious issue to consider when dealing with monitoring and controlling possible cases of infections during the post-pandemic period.

SARS-CoV-2 antibody testing has proven indispensable during the initial management of the COVID-19 pandemic and laboratory-based ELISAs and point-of-care rapid tests have been developed based on the detection of different viral SARS-CoV-2 antigens [1, 2]. The viral proteins that are used in this respect are the spike (S) protein containing the receptor-binding domain (RBD), located on the surface of the virus. In addition, also the nucleocapsid (N) protein, located in the inside of the viral particles, is commonly used for diagnostic purposes. Due to sufficient diagnostic accuracy values and medium- to high-throughput capabilities, these antibody tests are now routinely used to identify acute or previous SARS-CoV-2 infections [3–7]. Also, for the characterization of convalescent plasma either from original infections or determined post-vaccination antibody levels, such assays have been proven useful [8]. The determination of local seroprevalence patterns for immunoglobulins directed against SARS-CoV-2 antigens in afflicted populations remains therefore essential for containing the still ongoing pandemic.

In this respect, especially the seroprevalence of SARS-CoV-2, neutralizing antibodies and their local and temporal dynamics within a given population are essential for monitoring the overall immunity status. However, despite the fact that seroepidemiological data represent the only way to gain knowledge about the prevalence of asymptomatic and light symptomatic cases, such studies are still in short supply. This is even more surprising as also the potency and long-lasting effect of the acquired immune responses are not yet sufficiently accounted for, both of which are highly important from a public health perspective.

In the present study, we describe the analysis of seroprevalence data for IgG antibodies against SARS-CoV-2 S/RBD and N antigens for the federal state of Upper Austria (Austria) for a time frame of 4 months. Data have been obtained from self-referral individuals by using a commercial SARS-CoV-2 antibody rapid test from Genspeed Biotech GmbH in pharmacies distributed throughout the federal state of Upper Austria (Austria). The data described could serve as an example for establishing potential monitoring networks for (post-) pandemic surveillance of regionally defined populations. Examples include the development of the immune status over time for selected antigens and/or the detection of sufficiently high antibody levels that might qualify as indicative for a potential neutralizing activity according to the current WHO standard (NIBSC 20/136).

## Materials and methods

### Participants and recruitment

As with 2020, the population of Upper Austria consisted of 1,490,279 citizens (50.36% female, 49.64% male) (https://www.land-oberoesterreich.gv.at/statistik.htm). In 2021, Linz, the capital of Upper Austria, comprised 207,812 citizens (51.3% female, 48.7% male) (https://www.linz.at/zahlen). In the course of this study, we have obtained and analyzed the results of a serological screening campaign performed in pharmacies throughout the federal state of Upper Austria (Austria) from December 18, 2020, until April 15, 2021. However, we are aware that a certain bias is inevitably introduced by this approach since we are dealing with non-random sampling in the sense of self-referral of individuals to the respective test sites. We have addressed this fact accordingly in the “Results” and “Discussion” sections.

### Ethics approval

The local ethics committee reviewed the use of data obtained through analysis of plasma samples from self-referral individuals for this analysis. It was determined that the research does not involve human subjects as the samples were de-identified and no link between samples and subjects exists. Therefore, it was concluded that no ethics vote was required.

### The multiplex micro-ELISA system

The commercial SARS-CoV-2 antibody rapid test from Genspeed Biotech GmbH (COVID19 IgG xPOC) was used to detect IgG antibodies against full length spike protein (S), the RBD of the spike protein as well as the nucleocapsid antigen (N) for SARS-CoV-2 (https://www.genspeed-biotech.com). The test is a chemiluminescence-based multiplex micro-ELISA (μELISA) system in a point-of-care format and analyzes blood obtained from a simple finger-prick sample. The outstanding feature of the microfluidic chip architecture is the ability to facilitate a sequential capillary force–driven liquid flow of reagents. The chip itself is designed with a circular inlet port (4 mm diameter) that interconnects with a microfluidic reaction channel (2 mm width; 23 mm length; 0.1 mm height) and the waste reservoir (20 mm width; 25 mm length; 0.6 mm maximum height). This architecture allows dispensing microliter volumes of different reagents to the inlet port sequentially which are immediately transported into the reaction channel through capillary forces. As soon as the inlet port is empty, the flow stops due to the surface tension at the proximal part of the reaction channel. When the next assay reagent is added, the flow resumes and the reagent in the reaction channel is displaced pushing the previous solution into the waste area.

Pre-analytics include the removal of erythrocytes by a simple filtration step before a drop of the sample is applied to the inlet port of the chip. After 2 min of sample incubation, another drop of biotinylated anti-human IgG detection antibody solution is added to the assay chip. Subsequently, the GENSPEED R2 analyzer sequentially and fully automatically dispenses all other reagents required to perform the multiplex micro-ELISA on the chip via micro-piezo pumps (mp6, Bartels-Mikrotechnik, Dortmund, Germany). Results are available within 15 to 20 min and a performance evaluation study in terms of detecting SARS-CoV-2 antigens has already been published demonstrating sensitivity values of 100% and specificity values of 93% [7].

The test system is based on proprietary technology combining microfluidic test chips with integrated microarrays, miniaturized optoelectronics, and automation of the assay procedure. The analyzer device itself is controlled and powered via the USB port of a tablet computer. Modular, Java-based software intuitively guides the user through the entire testing process.

The test chip is composed of an injection molded top part containing the microfluidic structures and a bottom foil (both polystyrol) onto which the respective antigens have been deposited. This homogeneous immobilization of the different viral antigens in the corresponding capture areas (1 × 2 mm) was accomplished using a sciFLEXARRAYER S12 (SCIENION AG, Germany) by printing a pattern of spots (diameter 200 μm, pitch 500 μm) with 4 columns and 2 lines using proprietary printing buffers. Production processes for spike protein (S), receptor-binding domain (RBD), and nucleocapsid protein (N) were developed at the University of Natural Resources and Life Sciences Vienna and eventually established for commercial production at enGenes Biotech (http://www.engenes.cc/) as described previously [9]. Finally, the microfluidic top part and the bottom foil with the immobilized viral antigens are connected by ultrasonic welding.

A major advantage in terms of time requirement for the analytical process is the abovementioned microfluidic architecture of the test chip, which enables capillary force–driven fluid flow of sample and reagents through the reaction channel. In particular, the shear forces induced by the reaction channel’s low height of only 100 μm reduces the time required for diffusion of the involved components by increasing the likelihood of biomolecular interactions between the analytes and the immobilized capture probes as they pass through the channel [10]. In contrast to laboratory-based ELISAs with turnaround times of up to 2 to 4 h, this microfluidics-based platform allows for assay procedures to be completed within only 15 to 20 min.

For data readout, the detection of the photons generated by a chemiluminescence reaction is achieved by using a custom photodiode array placed below the reaction channel of the test chip. The one-dimensional detector array with a spatial resolution of 1 mm is composed of 32 pixels. It features a low-noise equivalent power (10^−15^ W/Hz^1/2^) and is used in combination with an appropriate analog digital converter to integrate chemiluminescence signals over time (150 s integration time) yielding high signal-to-noise ratios.

Standard multiplexing assays for the detection of different biomarkers or pathogens in a sample in one test run have already been successfully demonstrated, e.g., DNA/RNA-based assays for the detection of genes encoding antibiotic resistance traits [11]. As a practical example for an already commercially available application of a protein-based assay, the current study on the detection of SARS-CoV-2-specific IgGs in different districts of the province of Upper Austria is presented in this study. Data regarding the analytical sensitivity of this assay have been established previously via determination of the limit of detection (LOD) using dilution series of reference antibodies (anti-2019-nCoV spike protein #C19S1-61H-20 and anti-2019-nCoV N-protein # C19NP-60H-20, SignalChem, Canada). The different LODs were confirmed with 20 replicate measurements each. The achieved LOD values for IgG antibodies against spike protein (S) and receptor-binding domain (RBD) were determined to be 12 ng/ml each, while the LOD for IgG antibodies against nucleocapsid protein (N) is even lower with 1.3 ng/ml (MS—personal communication). The described multiplex micro-ELISA system clearly outperforms standard lateral flow tests regarding multiplexing capabilities in terms of assay quality aspects. However, lateral flow strip tests are still often convincing with low production costs due to established high-throughput roll-to-roll manufacturing processes. In that regard, new roll-to-roll production processes also for foil-based microfluidics will allow a paradigm shift as has been demonstrated already by other experts in the field and is also driven by current research like, e.g., the NextGenMicrofluidics project (https://www.nextgenmicrofluidics.eu/) [12].

### Data processing and statistical analysis

The assay from Genspeed Biotech GmbH used in the course of this study provides a readout in units of mean chemiluminescence intensity values. For our data analysis, we assigned samples that showed positive signals for both S/RBD and N antigens to the group that has encountered a previous SARS-CoV-2 infection. On the other hand, samples that showed only a positive signal for S/RBD antigen were assigned to the group of vaccinated individuals. Data have been analyzed with the software package MATLAB (version R2020b). Faulty data entries due to missing values have been removed automatically during a data sanity check. We binned the tests corresponding to their geographical origin by comparing the ID number of the testing equipment to its registered location of the respective pharmacies in Upper Austria provided by the manufacturer.

### Public available data sets

For data analysis and comparison purposes, public available data sets including PCR-test results, vaccination rates, and incidence values have been used from the following sources: the COVID-19 dashboard from the Austrian Agency for Health and Food Safety (https://covid19-dashboard.ages.at/) and data supplied by the federal state of Upper Austria (“Datenquelle: Land Oberösterreich—data.ooe.gv.at”) (https://www.land-oberoesterreich.gv.at/119786.htm).

## Results

### Regional distribution of positive test results in the federal state of Upper Austria

**Fig. 1:**
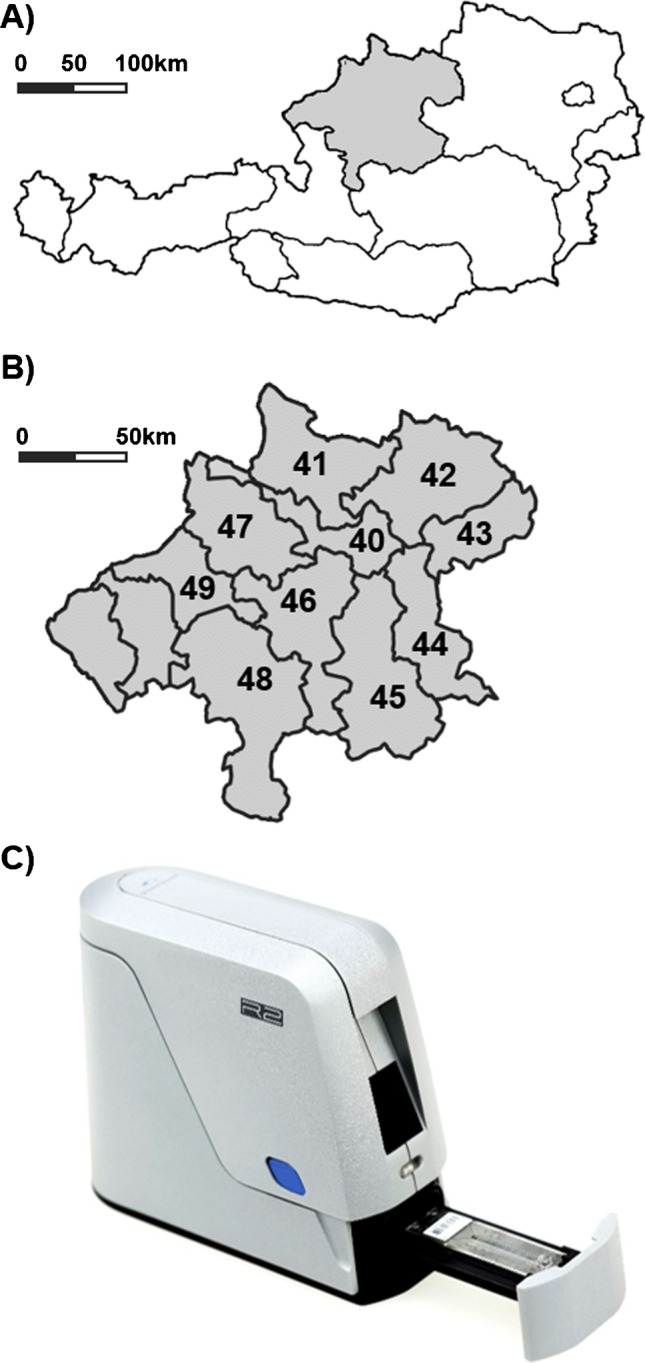
**A** Map of Austria with the federal district of Upper Austria depicted in grey. **B** Postal area codes for the individual districts of Upper Austria where measurements have been performed. **C** Measurement device and microfluidic test chip used in the course of this study (for a detailed description see the “Materials and methods” section)

The federal state of Upper Austria is divided into 10 postal code areas as depicted below (Fig. 1A and B). Approximately 7500 measurements have been performed in 38 local pharmacies throughout Upper Austria within 4 months (Table 1). Individuals appeared through self-referral and their immune status concerning immunoglobulin G reactive to different SARS-CoV-2 antigens was determined using the COVID19 IgG xPOC assay (Genspeed Biotech GmbH, Rainbach, Austria) (Fig. 1C).

**Table 1:** Number of participating pharmacies and total numbers of test performed per postal code area of Upper Austria

Postal area code	Number of participating pharmacies	Total numbers of test performed	Number of IgG-positive tests
40	7	1605	472
41	2	532	206
42	3	734	238
43	2	383	109
44	1	291	90
45	4	1113	332
46	9	1218	368
47	1	440	169
48	7	784	256
49	2	454	102

### The proportion of samples from infected individuals negatively correlates with the increasing vaccination rate in Upper Austria

**Fig. 2:**
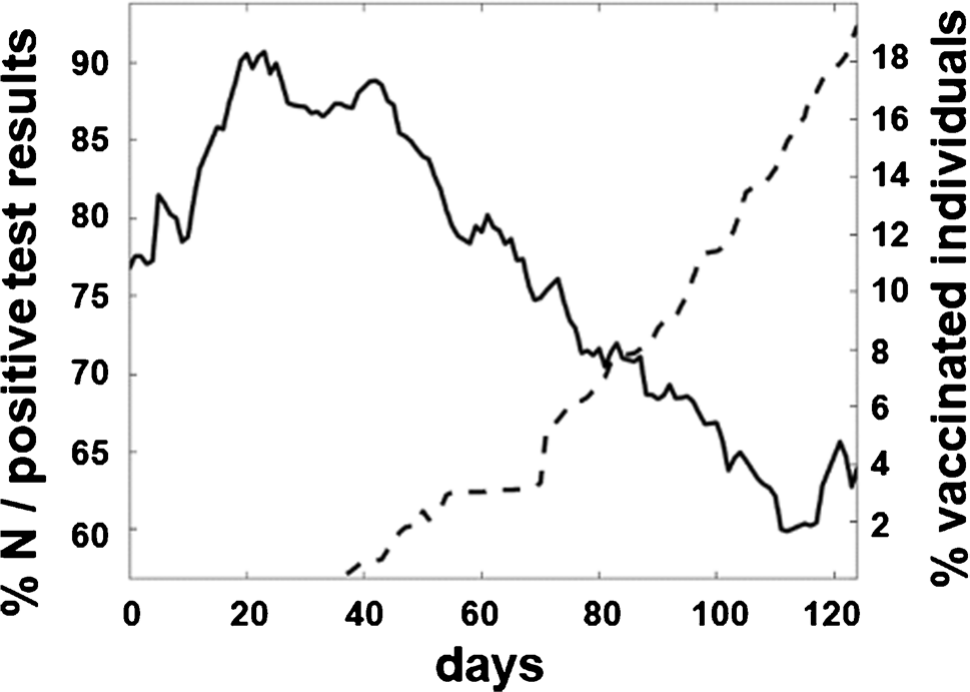
Decrease in the proportion of SARS-CoV-2 N antigen detected in positive antibody tests (solid line) and comparison to the vaccination rate in Upper Austria during a 4-month period (dashed line). Data are presented as a moving average over a 2-week period

There was a continuous decrease in the number of COVID-19-infected individuals during the period of our analyses (Fig. 2, solid line). Furthermore, we have observed an indirect correlation with the national vaccination rate (Fig. 2, dashed line). As the vaccination rate increased, a decrease in the number of infected participants was detected.

### Regional differences in the postal code areas of the federal state of Upper Austria

**Fig. 3:**
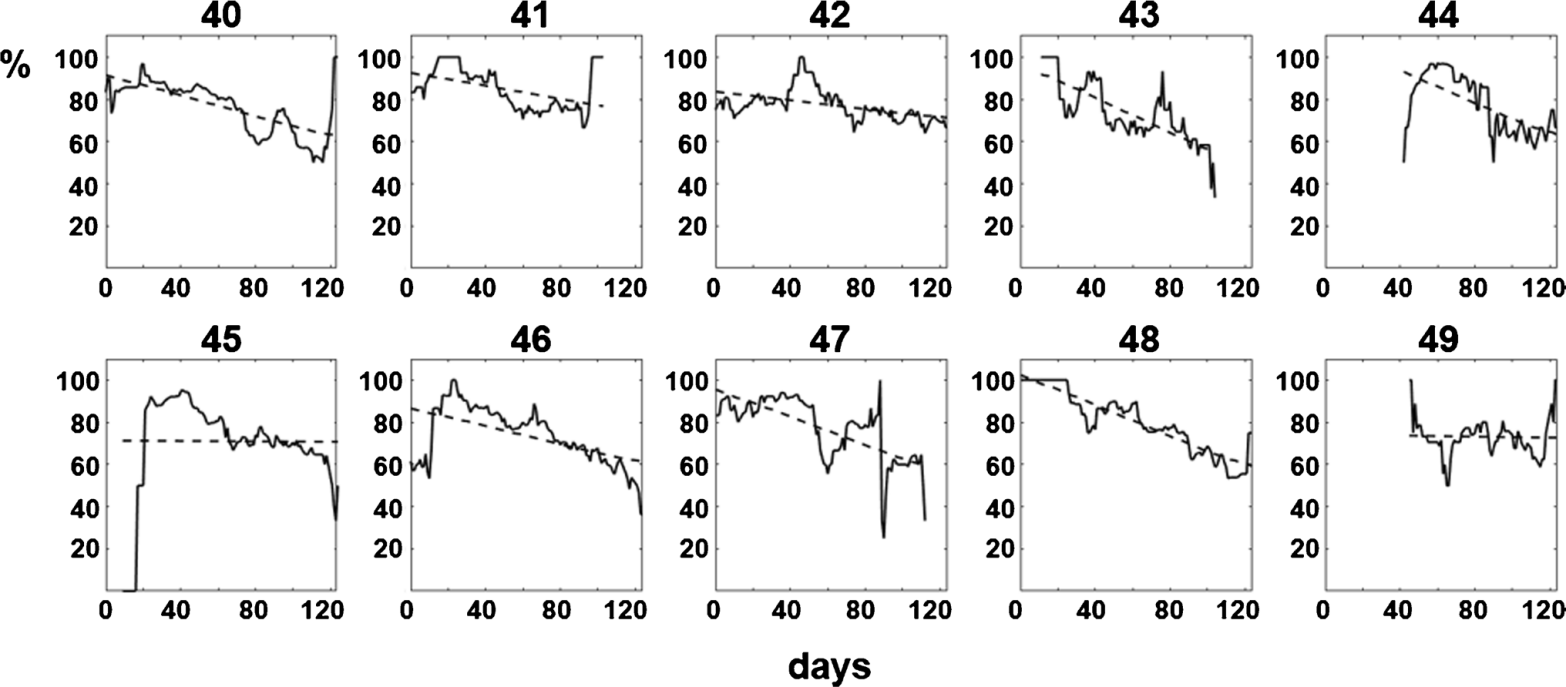
Decrease in the proportion of SARS-CoV-2 N antigen detected in positive antibody tests during a 4-month period for the individual postal code areas in Upper Austria (solid lines). Trends are indicated by dashed lines. Data are presented as a moving average over a 2-week period

In addition, we have also analyzed these data for the different postal code areas in Upper Austria during the 4-month period of our analysis (Fig. 3). As can be seen, there are differences concerning the regional trends of infection rates (depicted as a dashed line) as certain areas exhibit only a very slight decrease of the infection rates despite the launched vaccination programs.

### Presence of predicted protective antibodies according to WHO reference standard

**Fig. 4:**
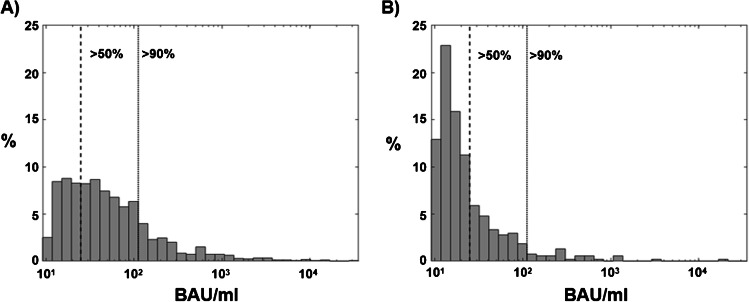
Presence of antibody levels with predicted protective activity from naturally infected and vaccinated study participants, respectively. Distribution of the obtained signal intensities of IgG-positive tests converted to BAU/ml according to WHO standard. Proportion on the right side of the dashed and the dotted lines indicate individuals harboring antibodies with > 50% or > 90% predicted protective activity, respectively. **A** Results obtained from naturally infected individuals (S/RBD- and N-positive). **B** Results from vaccinated individuals (S/RBD-positive)

According to the actual WHO reference standard (NIBSC 20/136), the limit for a positive test result for the Genspeed Biotech GmbH assay used in the course of this study was set at 10.7 binding antibody units (BAU)/ml based on correlation with a recently published study describing the persistence of neutralizing antibodies following an infection with SARS-CoV-2 [13]. Samples that exhibited a signal stronger than 24.9 BAU/ml are predicted to confer a protective effect of the induced antibodies against viral infection of > 50% (Fig. 4, dashed line). This value increases to > 90% when samples exhibited a signal stronger than 111.8 BAU/ml (Fig. 4, dotted line). Within the group of individuals that have experienced a natural infection (S/RBD- and N-positive samples), 60.89% and 17.67% exhibited a predicted protective effect of the induced antibodies of > 50% and > 90%, respectively (Fig. 4A). In the vaccinated group (S/RBD-positive), these values amount to 27.12% and 5.54% (Fig. 4B).

## Discussion

**Fig. 5:**
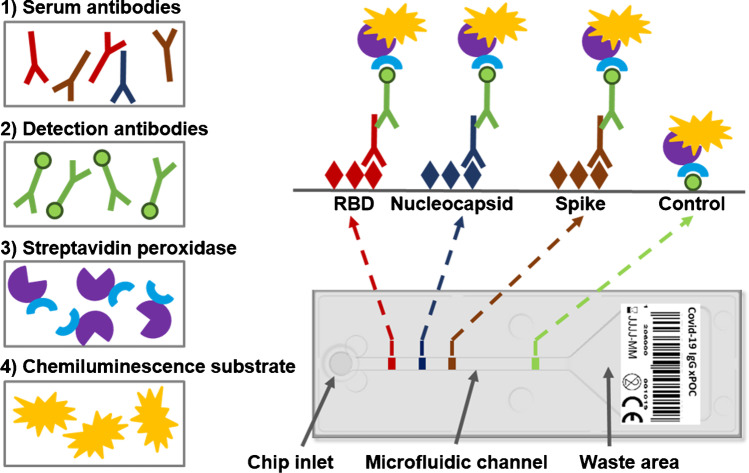
Graphical presentation of the measurement workflow. A capillary blood sample containing serum antibodies (1) is applied to the chip inlet and transported via capillary flow into the microfluidic channel, where these antibodies bind to the respective antigens (RBD, nucleocapsid, spike). After a brief incubation period, bound antibodies are labeled through the addition of biotinylated detection antibodies (2). Following the automatic, subsequent addition of enzyme solution (3), wash buffers, and chemiluminescence substrate reagent (4), the generated photons are quantified on a photodiode array and results are displayed following automated data analysis

The open design of the point-of-care rapid test from Genspeed Biotech GmbH (Rainbach, Austria) should make it possible to adapt virtually any existing laboratory ELISA to point of care level with a typical turnaround time of less than 30 min (Fig. 5). ELISAs for the detection of a single analyte such as procalcitonin or C-reactive protein have already been successfully transferred to this system and detection limits in the range of only 10 pg/ml could be achieved (MS—personal communication). However, since up to 10 positions in the microfluidic channel of the test chip can be used to immobilize capture molecules such as antibodies or antigens, simultaneous detection of different analytes is also possible via multiplexing. The individual positions in the microfluidic reaction channel can be used for the identification and quantification of up to 8 different biomarkers in a micro-ELISA setup with 2 positions normally reserved for controls. Alternatively, the individual positions can also be used to combine different ELISA methods such as sandwich-based or competitive assays for a single biomarker. This allows for enormously extending the dynamic range for the detection of a single marker compound.

In the current study, we have evaluated the presence of IgG antibodies directed against SARS-CoV-2 antigens from self-referred individuals obtained measured with the test system from Genspeed Biotech GmbH in pharmacies throughout the federal state of Upper Austria (Austria). By analyzing approximately 7500 samples, a prevalence value of roughly 30% was detected in this study group. We are aware that self-referral might introduce some bias within this analysis since people at risk for exposure or with medical health preconditions are more likely to have their antibody levels tested. However, we feel that for a general estimation of the seroprevalence in a given population, the sheer number of test results used for analysis should compensate for this limitation. In addition, such an approach represents a more life-like testing situation compared to clinical trials. The latter ones have to adhere to strictly regulated testing regimes and represent an optimal scenario that can hardly be achieved in reality.

In a first analysis, we examined the effect of the vaccination program against SARS-CoV-2 launched in Austria at the end of December 2020. As demonstrated in Fig. 2, a strong correlation can be seen between the decline of S/RBD- and N-positive test results (indicative of naturally infected individuals) and S/RBD-only-positive test results (result of a successful vaccination). Although there are reports that also naturally infected individuals are sometimes exhibiting only a very weak positive N signal, we feel that these data are highly encouraging and are a clear demonstration of the benefit of national vaccination approaches. In Fig. 3, we have analyzed these data on the level of the individual postal code areas in Upper Austria and have largely confirmed the previous results. However, while for most of the areas the trend mimics the results seen in Fig. 2, some areas are exhibiting only a very weak correlation. This effect needs further attention since it might reflect a lower-than-average share of immunized individuals in these regions.

The current gold standard for SARS-CoV-2 serology are neutralization assays that detect the presence of antibodies that can neutralize the virus and inhibit the infection by blocking access to the ACE2 receptor on the target cell by binding to the receptor-binding domain (RBD) on the viral spike protein. A major drawback of these neutralization assays is that they are highly laborious and expensive and can only be performed in specialized biosafety level 3 laboratories. Related tests such as pseudo-typed neutralization assays are also very labor-intensive and not suitable for high-throughput analyses [14, 15]. To bypass these limitations, recent work from several groups has already succeeded to prove the hypothesis that the presence of antibodies that interfere with binding of the virus to the surface protein ACE2 in infected individuals is indeed positively correlating with an actual neutralizing activity. For example, a strong relationship has been demonstrated between the mean neutralization level and the protective efficacy for different vaccines—a 50% protective neutralization level is observed on average at about a fifth of the antibody titer found in convalescent plasma [16].

Additional reports have shown a significant correlation between the presence of spike protein binding antibodies and various forms of functional virus neutralization assays [17–23]. One study further characterized these observations and demonstrated that only antibodies specific for the receptor-binding domain of the spike protein (S/RBD) demonstrate an excellent correlation with before mentioned neutralization assays already during the early phase of an infection [9]. It is worth to mention that the detection of antibodies binding to the viral nucleocapsid (N) protein of SARS-CoV-2 does not always correlate with the presence of S/RBD-neutralizing antibodies and serology tests that rely only on the detection of N-protein might be misleading in this respect [6].

Resistance to infection with SARS-CoV-2 acquired through vaccination represents an impressive success story of biomedical research. Multiple studies have already reported that vaccination confers protection against reinfection and/or is able to reduce the risk of clinically significant outcomes. While patients that have recovered from past COVID-19 episodes have been estimated to demonstrate approximately 90% protection against reinfection [24], the values obtained through vaccination have been reported to lie in the range from 50 to 95% [25].

In light of these reports, we have also used the respective binding antibody units (BAU)/ml for wild-type SARS-CoV-2 according to WHO standard (NIBSC 20/136) from the data obtained in the course of this study [13]. Within the group of individuals that have experienced a natural infection (S/RBD- and N-positive), 60.89% exhibited a predicted protective effect of the induced antibodies against viral infection of > 50% (equivalent to a BAU/ml value of 24.9). Also, in this group, 17.67% of individuals exhibit a predictive protective effect of > 90% (equivalent to a BAU/ml value of 111.8).

On the other hand, the predicted protective effect of antibodies present in individuals following administration of one of the available vaccines (S/RBD-positive) was 27.12% for > 50% protection and 5.54% for > 90% protection, respectively. There are several reasons that can explain the lower numbers in vaccinated individuals seen in our analyses. For example, it has to be stressed that we have analyzed data that were obtained during the very beginning of the vaccination phase in Upper Austria. The target group at that time consisted mainly of aged individuals and people with pre-existing health conditions. Besides representing a group that might also respond not so well to the stimulation of the immune system by vaccination, most of these individuals will have obtained only the first dose of the respective vaccine at that time. In addition, it has previously also been demonstrated that different brands of vaccines are inducing varying levels of circulating antibodies that might also be reflected in our sample data. A follow-up study to characterize the presence of protective antibodies after completing a full vaccination program is therefore highly warranted.

## Conclusion

In the present report, we have demonstrated the benefits of seroepidemiological testing for the determination of the immunization status of a population in a given region through a commercial SARS-CoV-2 antibody rapid test from Genspeed Biotech GmbH. By identifying individuals that have obtained protective antibodies through natural infection as well as through vaccination, any change in seroprevalence values in a population could be used as an indicator to launch or intensify, e.g., local testing and/or vaccination efforts. Therefore, establishing national testing networks offering low-threshold access opportunities will generate a valuable additional effect when dealing with the challenges of the post-pandemic times ahead.
